# cpRAS: a novel circularly permuted RAS-like GTPase domain with a highly scattered phylogenetic distribution

**DOI:** 10.1186/1745-6150-3-21

**Published:** 2008-05-29

**Authors:** Marek Elias, Marian Novotny

**Affiliations:** 1 Charles University in Prague, Faculty of Science, Department of Botany, Benatska 2, 128 01 Prague 2, Czech Republic; 2 Charles University in Prague, Faculty of Science, Department of Cell Biology Vinicna 7, 128 44 Prague 2, Czech Republic

## Abstract

A recent systematic survey suggested that the YRG (or YawG/YlqF) family with the G4-G5-G1-G2-G3 order of the conserved GTPase motifs represents the only possible circularly permuted variation of the canonical GTPase structure. Here we show that a different circularly permuted GTPase domain actually does exist, conforming to the pattern G3-G4-G5-G1-G2. The domain, dubbed cpRAS, is a variant of RAS family GTPases and occurs in two types of larger proteins, either inserted into a region homologous to a bacterial group of proteins classified as COG2373 and potentially related to the alpha-2-macroglobulin family (so far a single protein in *Dictyostelium*) or in combination with a von Willebrand factor type A (VWA) domain. For the latter protein type, which was found in a few metazoans and several distantly related protists, existence in the common ancestor of opisthokonts, Amoebozoa and excavates followed by at least eight independent losses may be inferred. Our findings thus bring further evidence for the importance of parallel reduction of ancestral complexity in the eukaryotic evolution.

This article was reviewed by Lakshminarayan Iyer and Fyodor Kondrashov. For the full reviews, please go to the Reviewers' comments section.

## Findings

One of the evolutionary innovations that may affect a protein fold is the so-called circular permutation [1,2]. This term refers to a situation whereby the N-terminal part of one protein is homologous to the C-terminal part of another protein and *vice versa*. Circular permutations have been found in a wide range of proteins [3] and other examples are definitely to be discovered in the rapidly growing protein sequence databases. Some authors discriminate between a swap and a circular permutation [3,4]. The swap is then any mutually altered order of homologous regions in proteins, while the circular permutation refers to a special case of a swap where swapped regions cover essentially the whole protein length. We shall use the term "circular permutation" to describe an altered order of N- and C-terminal segments of individual proteins domains, regardless of the actual size and domain composition of the whole proteins.

P-loop GTPases and related proteins form a vast superclass of globular α/β proteins with the most conserved and functionally important regions denoted as G1 to G5 motifs [5,6]. One of the many distinct evolutionary lineages of the superclass referred to as YawG/YlqF [5] or YRG [7] family comprises proteins with a non-canonical order of the conserved motifs, which are arrayed in a circularly permuted fashion G4-G5-G1-G2-G3. However, crystal structures of several representatives [8,9] (Kniewel et al., unpublished, PDB accession number 1PUJ) and biochemical studies [7,10] have demonstrated a rather typical GTPase fold and the expected GTP-binding/GTPase function of these proteins.

A recent systematic search for circularly permuted GTPases has identified the YRG family as the only group with such a modification of the GTPase domain [11]. The authors argued that other possible circular permutations of the GTPase domain are unlikely owing to structural or functional constrains. For example, according to Anand and colleagues, the G3-G4-G5-G1-G2 permutation, which breaks the reverse turn between the strands β2 and β3, should impair the proper folding of the protein [11].

**Figure 1:**
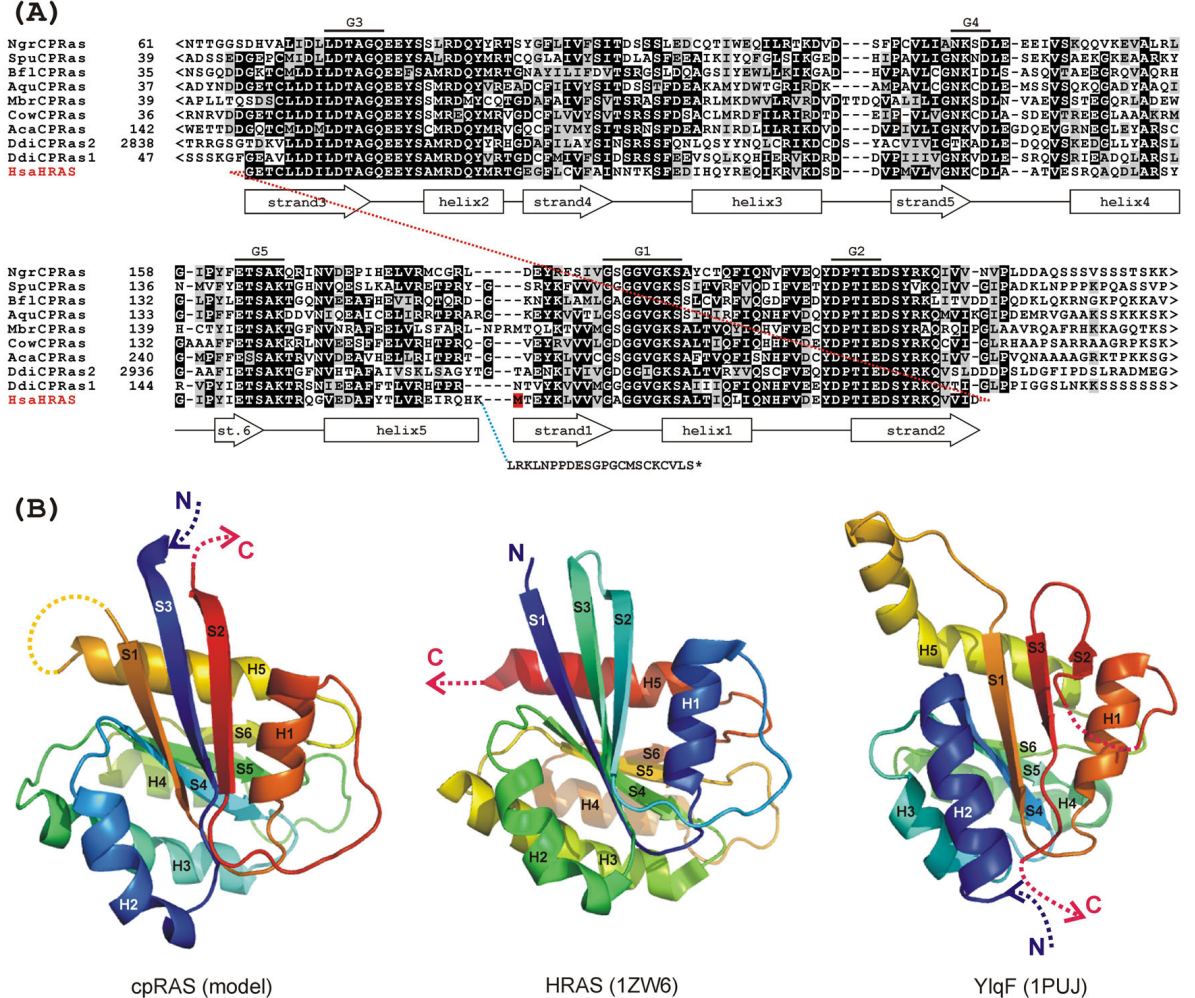
**(A) **Multiple sequence alignment of the cpRAS GTPase domains. The sequence of the canonical HRAS GTPase domain is included for comparison. The M residue with a red background is the actual N-terminus of the HRAS protein, the dashed red line indicates the peptide bond within HRAS disjoined so that it can be aligned with the cpRAS domain. The blue dashed line indicates a bond connecting the HRAS GTPase domain with an unstructured C-terminal tail. Strands and helices as experimentally determined for GDP-bound HRAS are indicated below the alignment. G1 to G5 above the alignment mark the five conserved GTPase signature motifs as defined in [5]. Species abbreviations: Aca – *Acanthamoeba castellanii*, Aqu – *Amphimedon queenslandica*, Bfl – *Branchiostoma floridae*, Cow – *Capsaspora owczarzaki*, Ddi – *Dictyostelium discoideum*, Hsa – *Homo sapiens*, Mbr – *Monosiga brevicollis*, Ngr – *Naegleria gruberi*, Spu – *Strongylocentrotus purpuratus*. For accession numbers of the protein sequences see Additional file 1. **(B) **Predicted tertiary structure of the cpRAS domain and its comparison with the canonical human HRAS GTPase domain and the circularly permuted GTPase domain of the *Bacillus subtilis *protein YlqF. Dashed arrows represent parts of the proteins located N- or C-terminally of the GTPase domain. The dashed line connecting the helix5 and strand1 in the cpRAS model indicates an expected loop between these two elements, although the precise structure of this part of the domain could not be modelled (see the text). The dashed line connecting the helix1 and strand2 in YlqF represents a putative loop that is missing from the solved structure. Secondary structure elements in cpRAS and YlqF are labelled according to the equivalent β-strands and α-helices (S1 to S6 and H1 to H5) in HRAS, although their actual position in the polypeptide chain is different. Colours of the spectrum (from violet blue to red) are assigned to consecutive parts of the structures starting from the N-terminus.

In the course of our systematic survey of the Ras superfamily, we noted that two large uncharacterised proteins from *Dictyostelium discoideum*, EAL68747.1 and EAL60755.1 (hereafter referred to as DdiCPRas1 and DdiCPRas2, respectively), contain a putative circularly permuted GTPase domain with the conserved motifs G1-G2 lying downstream of the G3-G4-G5 motifs (Fig. 1A). At the secondary structure level, the region corresponding to strand1-helix1-strand2 of a canonical GTPase domain (here exemplified by the human HRAS) directly follows the region equivalent to helix5. Based on sequence comparison with available GTPase sequences, the domain in DdiCPRas1 and DdiCPRas2 could be readily classified into the RAS family, which consists of mostly small proteins composed of a GTPase domain and a hypervariable C-terminal tail with a prenylated cysteine residue ensuring membrane localisation of the protein [12,13]. Except the circular permutation, the domains in DdiCPRas1 and DdiCPRas2 are rather typical RAS family GTPase domains including, for instance, the "TIE" G2 motif (Fig. 1A) characteristic for the family. DdiCPRas1 and DdiCPRas2 thus define a new subtype of RAS family proteins.

Using MODELLER 9.1 [14] and the human HRAS structure (1ZW6) as a template, we build a 3-D model of the cpRAS domain of DdiCPRas1 (Fig. 1B and Additional file 1). Because of the circular permutation, the DdiCPRas1 sequence had to be reshuffled so that it could have been aligned with HRAS and the N- and C-termini of the model were created artificially by removing the bond between the strands 2 and 3. The template did not help in elucidating the conformation of the putative loop between the helix 5 and strand 1, so this loop is not included in the model. Furthermore, it is likely that the actual appearance and position of the helix5 and strand1 is slightly different from that indicated by the model because of the connection between them implicated by the primary structure of the cpRAS domain. Nevertheless, although experimental verification is required, the model and the presence of typical GTPase signature motifs suggest that DdiCPRas1 very likely retains a GTPase function despite the circular permutation. The GTPase function is also conserved in the previously described version of a circularly permuted GTPase domain of the YRG family [7,10], which however differs from the cpRAS domain in lacking a connection between the strand3 and helix2 rather than between strands 2 and 3 (Fig. 1B).

**Figure 2:**
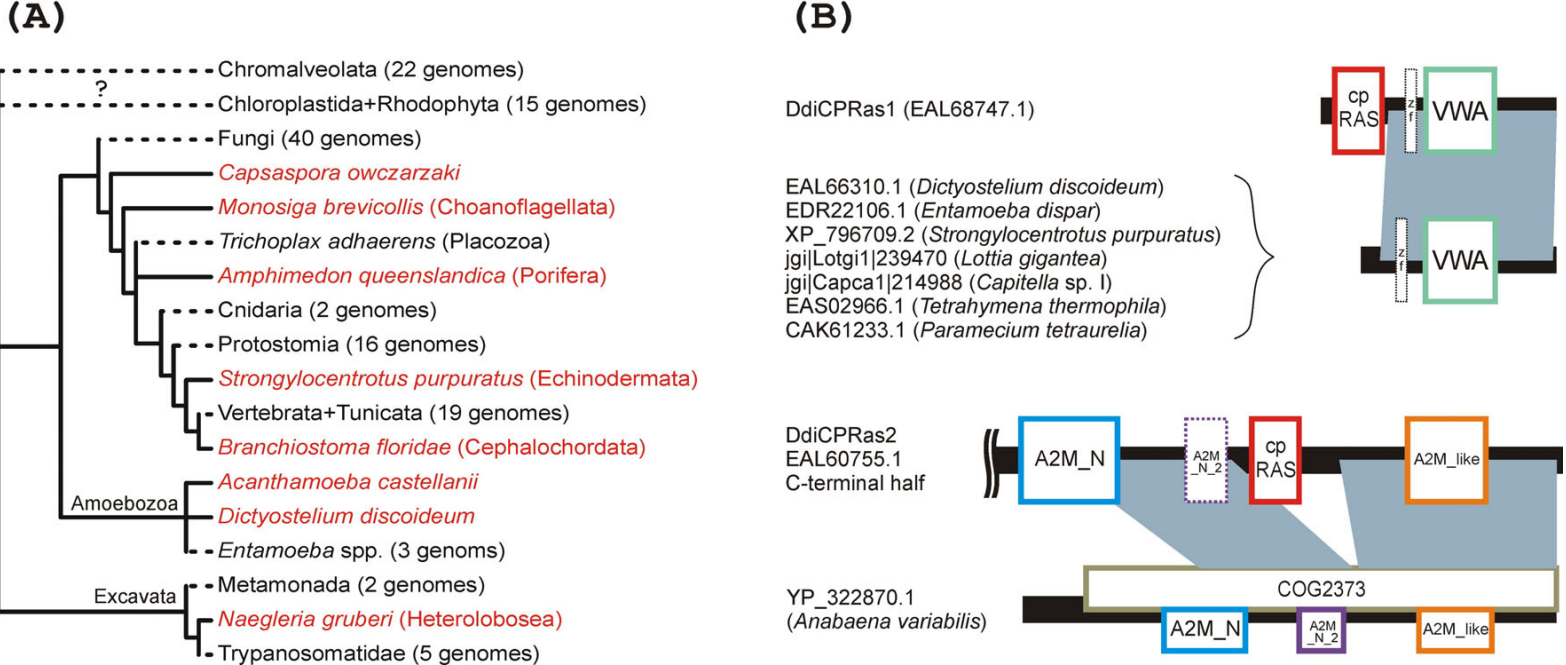
**(A) **Phylogenetic distribution and presumed losses of the cpRAS domain in eukaryotes. Species containing the domain (in red) are shown in the context of the global eukaryotic phylogeny (based on recent phylogenetic literature). Dashed branches correspond to lineages that seem to have lost the cpRAS domain (genomes examined for the presence of but lacking the domain are listed in Table S1 in Additional file 1). The question mark indicates that it is unclear whether the absence of the cpRAS domain in Chromalveolata and Chloroplastida+Rhodophyta is primary or secondary. **(B) **Domain organisation of CPRas proteins. Blue zones indicate regions of mutual homology between CPRas proteins (here exemplified by *Dictyostelium *representatives) and related proteins lacking a cpRAS domain indicated by BLASTP. Only the C-terminal half of DdiCPRas2 is shown (the N-terminal half does not contain any recognisable conserved domain or motif). The domains with dashed outlines are recognised with insignificant E-values or are suggested on the basis of visual inspection of a multiple alignment only. For brevity, only a single representative of a larger family of paralogous VWA domain-containing proteins is indicated for each *Tetrahymena *and *Paramecium*.

In order to illuminate the evolutionary origin of the cpRAS domain, we used BLAST [15] to search genome databases for orthologs of the *Dictyostelium *CPRas1 and CPRas2 proteins (see Additional file 1 for details on sequence data sources). Interestingly, the cpRAS domain was found in other species, but the phylogenetic distribution of cpRAS is highly scattered, comprising a few metazoans (so far only the lancet *Branchiostoma floridae*, the sea urchin *Strongylocentrotus purpuratus*, and the sponge *Amphimedon queenslandica*), the choanoflagellate *Monosiga brevicollis*, the unicellular opisthokont *Capsaspora owczarzaki*, the amoebozoan *Acanthamoeba castellanii*, and the heterolobosean *Naegleria gruberi *(Fig. 1A, 2A). According to their domain architecture, these proteins fall into two groups (Fig. 2B).

The first comprises the *Dictyostelium *DdiCPRas1 and CPRas from other species and is represented by proteins of ≈ 840–970 amino acid residues characterised by a poorly conserved N-terminal region of 40–66 residues without any recognisable homology to other proteins, the cpRAS domain, a poorly conserved putative linker region, and a conserved C-terminal half that contains a domain annotated as "von Willebrand factor (vWF) type A (VWA)" domain in the SMART collection [16] or as "Sec23/Sec24 trunk" domain (PF04811) in the Pfam database [17]. In addition, inspection of a multiple alignment suggests that a type of zinc finger motif including four absolutely conserved cysteine residues may be present in most of these proteins (except the protein from *Acanthamoeba*) upstream the VWA domain, although it receives only high E-values or is not recognised at all by domain identification tools. The CPRas protein from *Naegleria *additionally harbours a RING finger motif fused to the C-terminus. Interestingly, proteins remarkably similar to the C-terminal half of this CPRas type including the potential zinc finger but lacking even vestiges of an N-terminal cpRAS domain can be found in several taxa, namely *Dictyostelium*, *Entamoeba dispar*, some metazoans (the mollusc *Lottia gigantea*, the annelid *Capitella *sp. I, *Strongylocentrotus*), and ciliates (Fig. 2B). Based on the level of mutual similarity, these proteins appear to be a group of their own, evolutionarily separate from the C-terminal half of CPRas proteins with the cpRAS-VWA domain arrangement (data not shown).

The large *Dictyostelium *protein DdiCPRas2 (3933 residues) shares with other cpRAS proteins only the circularly permuted GTPase domain corresponding to residues 2844–3013, while the rest differs completely (Fig. 2B). The N-terminal part of the DdiCPRas2 shows no clear homology to other proteins or domains in searches with BLASTP, SMART, Search Pfam, or CD-search, but two large blocks of the protein flanking N- and C-terminally the cpRAS domain, approximately residues 2140–2771 and 3158 to the very C-terminus, show significant BLASTP similarity (E-values of 3.10–32 and higher) to a group of bacterial uncharacterised proteins (in Fig. 2B exemplified by a protein from *Anabaena variabilis*) representing the family COG2373 in the CDD database [18]. The family is named "Large extracellular alpha-helical protein" [19], but the basis for this annotation is unclear and should be treated with caution. This family may actually be homologous to the eukaryotic alpha-2-macroglobulin family of serum proteins, because a series of domain typical for this family, specifically the A2M_N (PF01835), A2M_N_2 (PF07703), and A2M_like (cd02891) domains, can be recognised in COG2373 proteins using Search Pfam and/or CD-search. These domains appear to be conserved also in DdiCPRas2, although the A2M_N_2 domain is recognised with insignificant E-value only. The unique and unusual architecture of DdiCPRas2 among other proteins with the cpRAS domain might raise doubts about the accuracy of the respective nucleotide sequence or gene model. However, an incomplete sequence of an obviously orthologous gene with potentially the same domain architecture is present in a low-coverage assembly of the genome of another *Dictyostelium *species, *D. purpureum *(scaffold_491 [20]), confirming the authenticity of the DdiCPRas2-like type of CPRas proteins.

According to our phylogenetic analysis based on an extensive sampling of the RAS family (168 sequences), all cpRAS domains are of a monophyletic origin with the cpRAS domain of DdiCPRas2 nested within the cpRAS clade, although without support from bootstrap analysis (data not shown). Given also its much wider phylogenetic distribution, it seems that the cpRAS-VWA domain organisation evolutionarily precedes the DdiCPRas2-like domain arrangement. Interestingly, when the DdiCPRas2 sequence excluding the cpRAS domain is used as a BLASTP query against the NCBI nr database, the significant hits comprise sequences from diverse bacteria but only one eukaryotic entry – another COG2373-like protein (but lacking a GTPase domain) from *Dictyostelium *(EAL60755.1). We therefore suggest that the type of CPRas proteins represented by DdiCPRas2, specifically its C-terminal part, evolved by insertion of a copy of a cpRAS domain from a cpRAS-VWA protein into a COG2373-like protein introduced into the *Dictyostelium *lineage by horizontal gene transfer (HGT) from prokaryotes.

The YRG family is widespread in all Eubacteria, Archaebacteria, and Eukaryota [6,7,11], so the circular permutation that generated the GTPase domain of this family probably occurred before the last universal common ancestor (LUCA). By contrast, the cpRAS domain is apparently a much later eukaryote-specific derivative of the GTPase fold. The scattered phylogenetic distribution of the cpRAS domain might indicate its origin in a particular eukaryotic lineage followed by dissemination by HGT. However, our phylogenetic analysis of the full cpRAS-VWA protein sequences (see Fig. S1 in Additional file 1) does not provide any strong evidence for HGT among the distinct eukaryotic lineages and the results are compatible with the scenario whereby cpRAS-VWA occurred already in the common ancestor of Excavata, Amoebozoa, and Opisthokonta but was lost independently from at least eight lineages (Fig. 2A). Although sequencing of additional genomes is necessary to test this hypothesis, it is likely that most cases of the absence of the cpRAS domain will indeed prove as resulting from secondary losses. The cpRAS domain thus appears to provide further evidence for an unexpected complexity of ancestral eukaryotes and the importance of secondary reduction in the eukaryotic evolution (see, e.g., [21,22]).

There appear to be no specific or well-defined functions defined for the domains that physically combine with the cpRAS domain (the VWA domain and the alpha-2-macroglobulin family domains), so the domain architecture of CPRas proteins provides little clues as to their possible cellular roles. Their highly scattered phylogenetic distribution in a disparate set of organisms indicates that they may be implicated in rather specialised processes. The CPRas proteins are thus definitely interesting candidates for experimental characterisation.

In summary, our findings correct the claim by Anand and colleagues [11] that the G4-G5-G1-G2-G3 arrangement of the conserved GTPase motifs is „the only possible circular permutation that can exist in nature" and further add to the structural variations of the GTPase domain. Future broader sampling of eukaryotic genomes will reveal more on the evolutionary origin of the cpRAS domain and functional studies will establish the cellular role of CPRas proteins.

## Competing interests

The authors declare that they have no competing interests.

## Authors' contributions

ME designed the study, collected and analysed the data and drafted the manuscript, MN performed the homology modelling and contributed to discussions and writing. Both authors read and approved the manuscript

## Reviewers' comments

### Reviewer 1: Lakshminarayan Iyer, National Center for Biotechnology Information, National Library of Medicine, National Institutes of Health, Bethesda, USA

This is a previously uncharacterized, circularly permuted subfamily of the Ras GTPases that is sporadically distributed in a wide phyletic range of eukaryotes. The observation merits publication in order to bring attention to this subfamily of proteins. I have a few points and several editorial suggestions.

Major comments

-The domain architectures need to be precisely described and defined. There should only be one standard name for domains. Sec23/Sec24 trunk is the vwA domain.

***Authors' response: ****These two names indeed designate the same domain. We have revised the respective part of the text so that this is now clearer. However, because the different names for this same domain are used variously in standard databases (SMART, Pfam), we believe it is appropriate to mention both*.

COG names are useful when the domain function or fold is not clear. In the case of COG2373, there is little doubt as to what it is and it should be correctly represented.

The alpha-2 macroglobulin domain needs to be more precisely described and it definitely is not alpha helical. The cpRAS domain is lodged within multiple repeats of the macroglobulin-like domain; this part is not described or analyzed correctly.

***Authors' response: ****It is true that COG2373 proteins and DdiCPRas2 share several domains with the alpha-2 macroglobulin family of serum proteins, so we have added details on this to the respective paragraph and to *Fig. 2B. *It is possible that COG2373 proteins are bacterial orthologs of the alpha-2 macroglobulin family, but the similarity is low and to our best knowledge, no COG2373 protein has been functionally characterised, so the actual nature of these proteins remains uncertain. In addition, the bacterial COG2373 proteins and DdiCPRas2 exhibit regions of homology extending beyond the alpha-2 macroglobulin domains and this fact is well captured by the concept of the COG2373 family as defined in the standard database of conserved domains and families – the CDD database. We have therefore kept the reference to the COG2373 family in the revised text, though with doubts expressed about the annotation of the family („Large extracellular alpha-helical protein")*.

Please check the architectures, some versions fused to the vWA have an additional ring finger.

***Authors' response: ****We are very thankful for this point, we indeed missed a RING finger motif at the C-terminus of the CPRas protein from *Naegleria gruberi. *In addition, further inspection of CPRas protein sequences revealed a possible zinc finger upstream the VWA domain. We have added a note on this into the revised text*.

- Given its solo presence, could DdiCPRas2 be an artificial fusion caused by misassembly? Such things are very common with genomic data. Please check and confirm.

***Authors' response: ****A protein with the same domain architecture is encoded by the genome of another *Dictyostelium – D. purpureum. *This gene was omitted from analyses described in the paper, because the sequence available at the moment is incomplete, but we have made a note on it in the revised manuscript, so the authenticity of DdiCPRas2 is now beyond any doubt*.

- Too much is being made of Anand et al's. statement about the existence of a single circularly permuted GTPase family.

***Authors' response: ****We follow the classical Popperian view on how science works. Anand et al. elaborated a hypothesis explaining why they had found only the G4-G5-G1-G2-G3 permutation of the GTPase domain (see also the expanded discussion on their claims incorporated into the revised manuscript upon suggestion of the second reviewer). This hypothesis should be taken seriously until evidence is found which would falsify it. This is exactly what our paper does*.

Circularly permuted proteins are observed across a wide range of protein folds and its emergence is solely determined by natural selection. I suggest that, it be mentioned earlier during the description of discovery of the circular permutation.

***Authors' response: ****We do not feel there is enough evidence supporting the notion that the emergence of circularly permuted proteins is solely determined by natural selection. We are rather inclined to the view that most such rearrangements are removed by negative selection and those that survive are mostly selectively neutral. However, it is true that circular permutations occur widely, so we have inserted a note on this into the first paragraph*.

- I don't see why pathogen defense needs to be invoked as a specialized process in which the cpRAS is involved and perhaps some elaboration is needed.

***Authors' response: ****The reviewer may be right in that the speculation on the potential involvement of CPRas proteins in pathogen defence is unsubstantiated. We have deleted the respective section from the revised manuscript and leave the possibilities on the function of CPRas proteins completely open*.

Other minor and editorial comments for the authors:

- In the description of the swap and the circular permutation, it should be clarified that the definitions are only for the protein domain and not the whole protein (which could have multiple domains)

***Authors' response: ****There is probably some misunderstanding here. The cited papers by the Unger's group *[3,4]*define the terms „circular permutations" and „swap" with respect to full proteins and make no reference to protein domains. We regard such definitions impractical, because they obscure the fact that domains are the actual basic units from which proteins are built. Following these definitions, the relationship between canonical GTPases (like HRAS) and CPRas proteins would be called „swap" without the understanding that it is nothing more than „circular permutations" of one particular domain (the GTPases domain). That's why we explicitly explain (see the last sentence of the first paragraph) that we apply the term „circular permutation" to the level of protein domains*.

-Last line of page 1:"..individual protein domains, regardless "of" the ..."

***Authors' response: ****The grammatical error has been corrected*.

- Shouldn't RAS be written as Ras?

***Authors' response: ****There is little consensus in the literature how the names of these GTPases should be written; all the forms „RAS", „Ras", or „ras" have been used in various sources, often with different meanings. We keep „RAS family" as a name for a broader group of GTPases comprising multiple subgroups as Ras, Rap, Rheb, CPRas etc. The name of the one specific representative of the family, the human HRAS, follows the standard HUGO nomenclature of human genes/proteins*.

- Page 3 last paragraph: Instead of opisthokont, use ichthyosporean as the latter is more precise.

***Authors' response: ****We do not think that it is more precise to treat *Capsaspora owczarzaki *as an ichthyosporean. It's true that it is classified in Ichthyosporea in the NCBI taxonomic scheme, but other authorities place the organism outside Ichthyosporea as a separate opisthokont lineage (e.g., Adl et al., J Euk Microbiol 2005, 52: 399–451). We therefore keep the designation „unicellular opisthokont" in the revised text*.

- Page 3. last paragraph: What is "global structure?", perhaps "domain architecture" is meant.

***Authors' response: ****Yes, we meant domain architecture. This expression is now used in the revised text, as it seems to be more appropriate*.

- Figure 2B. Revise according to the suggestions above.

***Authors' response: ****The figure has been revised accordingly*.

- I don't see the point in Table S1. I think the descriptions are clear enough.

***Authors' response: ****The text and the *Figure 1*mention only the whole eukaryotic clades for which evidence for the cpRAS domain is missing. We believe that it is useful to specify the actual species lacking the cpRAS domain, so we decided to retain the Table S1*.

### Reviewer 2: Fyodor Kondrashov, Section on Ecology, Behavior and Evolution, Division of Biological Sciences, University of California at San Diego, USA

This manuscript presents straightforward evidence in support of new RAS-like (cpRAS) domain with a distinct order of conserved GTPase motifs distinct from what has been claimed as the only possible arrangement found in YRG family of proteins. The manuscript also does a good job with exploring the phylogenetic patterns of the cpRAS domain and the possible functional implications. The phyletic pattern, which apparently involves multiple losses and horizontal gene transfers is intriguing, although is not unique. It would be interesting to see whether or not this domain could have evolved multiple times independently, however, this possibility is unlikely given that the circular permutation of the GTPase domains seems very similar between different species.

***Authors' response: ****We actually mention phylogenetic evidence for a single origin of the cpRAS domain in the text*.

It was not clear to me why the other authors decided that the YGR arrangement of the GTPase motifs was the only one possible. Perhaps a short discussion would be appropriate for the uninitiated readers.

***Authors' response: ****We have added to the third paragraph two sentences specifying the argument raised by Anand et al. against the existence of permutations other than G4-G5-G1-G2-G3*.

With this regard I would change the first sentence of the abstract to something like "A recent systematic survey claimed (or suggested) that the YRG (or YawG/YlqF) family with the G4-G5-G1-G2-G3 order of the conserved GTPase motifs represents the only possible circularly permuted variation of the canonical GTPase structure."

***Authors' response: ****We have modified the first sentence of the abstract following the reviewer's suggestion*.

## Supplementary Material

Additional file 1 **Supplementary Data**. A Microsoft Word document containing Supplementary Methods, Figure S1, Table S1, and supplementary sequence data. Click here for file
