# Supplementary material for: cpRAS: a novel circularly permuted RAS-like GTPase domain with a highly scattered phylogenetic distribution
Source: Biol Direct. 2008 May 29;3:21. doi: 10.1186/1745-6150-3-21 (PMC2430557; doi:10.1186/1745-6150-3-21)
Supplement: Additional file 1 — Supplementary Data. A Microsoft Word document containing Supplementary Methods, Figure S1, Table S1, and supplementary sequence data. [file 1745-6150-3-21-S1.doc]

**Supplementary Data to:**

### cpRAS: a novel circularly permuted RAS-like GTPase domain with a highly scattered phylogenetic distribution

Marek Elias^1*^ & Marian Novotny^2^

^1^Charles University in Prague, Faculty of Science, Department of Botany

Benatska 2, 128 01 Prague 2, Czech Republic

^*^Corresponding author

^2^Charles University in Prague, Faculty of Science, Department of Cell Biology

Vinicna 7, 128 44 Prague 2, Czech Republic

E-mail addresses:

ME: melias@natur.cuni.cz

MN: marian@natur.cuni.cz

This file contains:

1. Supplementary Methods
2. Figure S1
3. Table S1
4. Corrected and newly assembled sequences of CPRas proteins

##### **1. Supplementary Methods**

Sequences analysed in this study were identified by BLAST (Altschul et al., 1997) utilising sequence data from DDBJ/EMBL/GenBank (http://www.ncbi.nlm.nih.gov/), NCBI Trace Archive (http://www.ncbi.nlm.nih.gov/Traces/), Wellcome Trust Sanger Institute (http://www.sanger.ac.uk/), WUSTL Genome Sequencing Center (http://genome.wustl.edu/), DOE JGI (http://www.jgi.doe.gov/), and BCM HGSC (http://www.hgsc.bcm.tmc.edu/). Species with complete or high-coverage draft genome sequences that failed to reveal genes encoding a RAS-like circularly-permuted GTPase domain are listed in Table S1.

Complete sequences of the CPRas proteins from *Amphimedon queenslandica* and *Capsaspora owczarzaki* were assembled from whole genome shotgun (WGS) reads available in Trace Archive (ftp://ftp.ncbi.nih.gov/pub/TraceDB/). The assemblies were generated with the aid of the CAP3 program (Huang and Madan, 1999; http://pbil.univ-lyon1.fr/cap3.php).

Multiple alignments were constructed with the PROMALS web server (Pei et al., 2007; http://prodata.swmed.edu/promals/promals.php) and manually refined to account for the secondary structure of the closely related human HRAS structure (PDB accession number 1ZW6, Ford et al., 2006). Some gene models available in databases proved inaccurate upon visual inspection of multiple alignments; these were corrected by redefining the exon-intron structure to achieve a better fit of the resulting protein sequence with the alignment. The BoxShade 3.21 server (http://www.ch.embnet.org/software/BOX_form.html) was used to highlight the level of conservation of individual positions in the multiple sequence alignment at Fig. 1A.

Accession numbers of CPRas sequences analysed: BflCPRas – jgi|Brafl1|125403 (incorrect protein prediction; http://genome.jgi-psf.org/Brafl1/Brafl1.home.html); DdiCPRas1 and DdiCPRas2 – EAL68747.1 and EAL60755.1 (http://www.ncbi.nlm.nih.gov/), respectively; HsaHRAS – NP_005334.1 (http://www.ncbi.nlm.nih.gov/); MbrCPRas – jgi|Monbr1|20657 (incorrect protein prediction; http://genome.jgi-psf.org/Monbr1/Monbr1.home.html); NgrCPRas – jgi|Naegr1|68720 (incorrect protein prediction; http://genome.jgi-psf.org/Naegr1/Naegr1.home.html); SpuCPRas – XP_797936.2 (incorrect protein prediction; http://www.ncbi.nlm.nih.gov/). The CPRas protein from *Acanthamoeba castellanii* was predicted from the scaffold Contig18902_Contig6945_Contig21178 in the first assembly of the *Acanthamoeba* genome sequence (ftp://ftp.hgsc.bcm.tmc.edu/pub/data/AcastellaniNeff/Acas20080130/). Corrected and newly assembled and predicted protein sequences are inserted at the end of this document.

A homology model of the cpRAS domain of the DdiCPRas1 protein from *Dictyostelium discoideum* was build using the structure of the human HRAS (PDB accession number 1ZW6; Ford et al., 2006). An alignment of these two proteins was carried out using the PROBCONS web service (Do et al., 2005) and manually edited. The MODELLER 9.1 program (Sali and Blundell, 1993) was used to build a 3-D model of DdiCPRas1. The quality of the final model was checked using WhatCheck programs (Rodriguez et al., 1998).

Phylogenetic analyses were conducted with the maximum likelihood method implemented in PhyML-aLRT (Anisimova and Gascuel, 2006) using the WAG+Γ+I model (8 rate categories, all parameters estimated from the data) and protein multiple alignments excluding ambiguously aligned positions and positions containing gaps in most sequences. The approximately unbiased (AU) test of alternative tree topologies (Shimodaira, 2002) was done with the CONSEL (V0.1i) package (Shimodaira and Hasegawa, 2001) using site-wise likelihoods computed with PhyML-aLRT.

### *References to Supplementary Methods*

Altschul SF, Madden TL, Schäffer AA, Zhang J, Zhang Z, Miller W, Lipman DJ. (1997) Gapped BLAST and PSI-BLAST: a new generation of protein database search programs. *Nucleic Acids Res*, **25**:3389-402.

Anisimova M, Gascuel, O. (2006) Approximate likelihood ratio test for branchs: A fast, accurate and powerful alternative. *Syst Biol*, **55**:539-52.

Do CB, Mahabhashyam MS, Brudno M, Batzoglou S. (2005) ProbCons: Probabilistic consistency-based multiple sequence alignment. *Genome Res*, **15**:330-40.

Ford B, Hornak V, Kleinman H, Nassar N. (2006) Structure of a transient intermediate for GTP hydrolysis by ras. *Structure*, **14**:427-36.

Huang X, Madan A. (1999) CAP3: A DNA sequence assembly program. *Genome Res*, **9**, 868-77.

Pei J, Kim BH, Tang M, Grishin NV. (2007) PROMALS web server for accurate multiple protein sequence alignments. *Nucleic Acids Res*, **35**:W649-52.

Rodriguez R, Chinea G, Lopez N, Pons T, Vriend G. (1998) Homology modeling, model and software evaluation: three related resources. *Bioinformatics*, **14**:23-528.

Sali A, Blundell TL. (1993) Comparative protein modelling by satisfaction of spatial restraints. *J Mol Biol*, **234**:779-815.

Shimodaira H. (2002) An approximately unbiased test of phylogenetic tree selection. *Syst Biol*, **51**:492-508.

Shimodaira H, Hasegawa M. (2001) CONSEL: for assessing the confidence of phylogenetic tree selection. *Bioinformatics*, **17**:1246-1247.

**
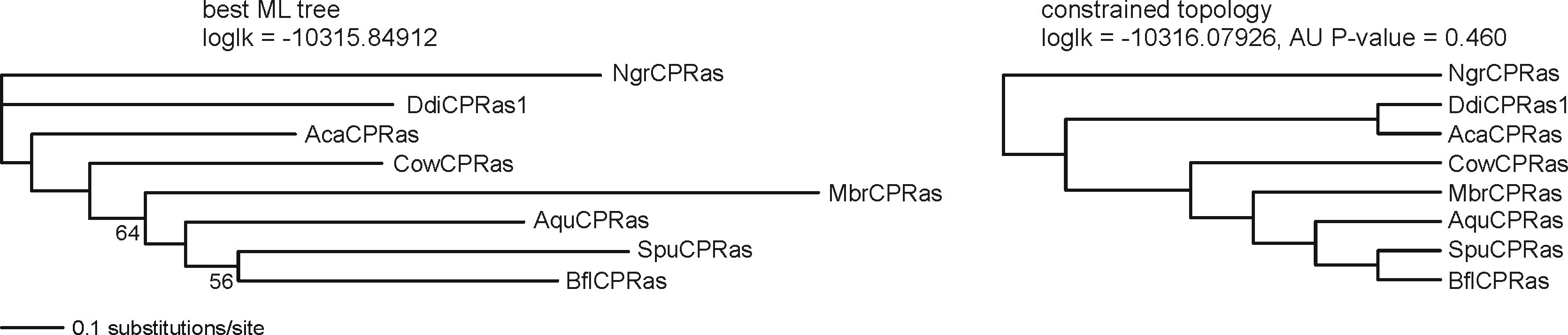
**

**2. Figure S1 – A phylogenetic analysis of CPRas proteins with the cpRAS-VWA domain arrangement.**

The tree on the left represents the ML tree as inferred by PhyML-aLRT (bootstrap values calculated from 100 replicates, only values >50% are shown). In this tree the proteins from *Dictyostelium discoideum* and *Acanthamoeba castellanii* do not form a monophyletic group, as would be expected from the known relationship of these species. However, a tree with a topology following the species tree (on the right) cannot be rejected as statistically worse by the AU test (P-value 0.460), hence vertical inheritance of the cpRAS-VWA proteins without the need to invoke horizontal gene transfer remains a plausible scenario. Species abbreviations as for Fig. 1A.

**3. Table S1** – Species with complete or draft genome sequences without discernible CPRas proteins.

The delimitation of clades of species corresponds to the clades shown at Fig. 2A in black.

| Clade | Species | Source of the genome sequence |
| --- | --- | --- |
| Tunicata  +Vertebrata | *Homo sapiens* | DDBJ/EMBL/GenBank |
|  | *Pan troglodytes* | DDBJ/EMBL/GenBank |
|  | *Macaca mulatta* | DDBJ/EMBL/GenBank |
|  | *Rattus norvegicus* | DDBJ/EMBL/GenBank |
|  | *Mus musculus* | DDBJ/EMBL/GenBank |
|  | *Canis familiaris* | DDBJ/EMBL/GenBank |
|  | *Bos taurus* | DDBJ/EMBL/GenBank |
|  | *Monodelphis domestica* | DDBJ/EMBL/GenBank |
|  | *Gallus gallus* | DDBJ/EMBL/GenBank |
|  | *Anolis carolinensis* | DDBJ/EMBL/GenBank |
|  | *Xenopus tropicalis* | JGI DOE, http://genome.jgi-psf.org/Xentr4/Xentr4.home.html |
|  | *Takifugu rubripes* | DDBJ/EMBL/GenBank |
|  | *Tetraodon nigroviridis* | DDBJ/EMBL/GenBank |
|  | *Danio rerio* | DDBJ/EMBL/GenBank |
|  | *Gasterosteus aculeatus* | DDBJ/EMBL/GenBank |
|  | *Oryzias latipes* | DDBJ/EMBL/GenBank |
|  | *Ciona intestinalis* | DDBJ/EMBL/GenBank |
|  | *Ciona savignyi* | DDBJ/EMBL/GenBank |
|  | *Oikopleura dioica* | Genoscope, http://www.genoscope.cns.fr/externe/GenomeBrowser/Oikopleura/ |
| Protostomia | *Drosophila melanogaster* | DDBJ/EMBL/GenBank |
|  | *Anopheles gambiae* | DDBJ/EMBL/GenBank |
|  | *Aedes aegypti* | DDBJ/EMBL/GenBank |
|  | *Tribolium castaneum* | DDBJ/EMBL/GenBank |
|  | *Nasonia vitripenis* | DDBJ/EMBL/GenBank |
|  | *Apis mellifera* | DDBJ/EMBL/GenBank |
|  | *Acyrthosiphon pisum* | DDBJ/EMBL/GenBank |
|  | *Daphnia pulex* | DDBJ/EMBL/GenBank |
|  | *Caenorhabditis elegans* | DDBJ/EMBL/GenBank |
|  | *Caenorhabditis briggsae* | DDBJ/EMBL/GenBank |
|  | *Brugia malayi* | DDBJ/EMBL/GenBank |
|  | *Lottia gigantea* | JGI DOE, http://genome.jgi-psf.org/Lotgi1/Lotgi1.home.html |
|  | *Helobdella robusta* | JGI DOE, http://genome.jgi-psf.org/Helro1/Helro1.home.html |
|  | *Capitella* sp. I | JGI DOE, http://genome.jgi-psf.org/Capca1/Capca1.home.html |
|  | *Schistosoma mansoni* | WTSI, http://www.sanger.ac.uk/Projects/S_mansoni/ |
|  | *Schmidtea mediterranea* | DDBJ/EMBL/GenBank |
| Cnidaria | *Nematostella vectensis* | DDBJ/EMBL/GenBank |
|  | *Hydra magnipapillata* | JCVI, https://research.venterinstitute.org/files/hydra_magnipapillata_WGS_assembly |
| Placozoa | *Trichoplax adhaerens* | JGI DOE, http://genome.jgi-psf.org/Triad1/Triad1.home.html |
| Fungi | *Phanerochaete chrysosporium* | DDBJ/EMBL/GenBank |
|  | *Postia placenta* | JGI DOE, http://genome.jgi-psf.org/Pospl1/Pospl1.home.html |
|  | *Laccaria bicolor* | DDBJ/EMBL/GenBank |
|  | *Coprinopsis cinerea* | DDBJ/EMBL/GenBank |
|  | *Cryptococcus neoformans* | DDBJ/EMBL/GenBank |
|  | *Ustilago maydis* | DDBJ/EMBL/GenBank |
|  | *Malassezia globosa* | DDBJ/EMBL/GenBank |
|  | Puccinia graminis | Broad Institute Puccinia graminis f. sp. Tritici Database annotation release 2http://www.broad.mit.edu/annotation/genome/puccinia_graminis |
|  | *Schizosaccharomyces pombe* | DDBJ/EMBL/GenBank |
|  | *Schizosaccharomyces japonicus* | DDBJ/EMBL/GenBank |
|  | *Yarrowia lipolytica* | DDBJ/EMBL/GenBank |
|  | *Debaryomyces hanseni* | DDBJ/EMBL/GenBank |
|  | *Candida albicans* | DDBJ/EMBL/GenBank |
|  | *Candida tropicalis* | DDBJ/EMBL/GenBank |
|  | *Lodderomyces elongisporus* | DDBJ/EMBL/GenBank |
|  | *Saccharomyces cerevisiae* | DDBJ/EMBL/GenBank |
|  | *Vanderwaltozyma polyspora* | DDBJ/EMBL/GenBank |
|  | Eremothecium gossypii | DDBJ/EMBL/GenBank |
|  | *Kluyveromyces lactis* | DDBJ/EMBL/GenBank |
|  | *Neurospora crassa* | DDBJ/EMBL/GenBank |
|  | *Gibberella zeae* | DDBJ/EMBL/GenBank |
|  | *Magnaporthe grisea* | DDBJ/EMBL/GenBank |
|  | Phaeosphaeria nodorum | DDBJ/EMBL/GenBank |
|  | *Sclerotinia sclerotiorum* | DDBJ/EMBL/GenBank |
|  | *Hypocrea virens* | DDBJ/EMBL/GenBank |
|  | *Aspergillus fumigatus* | DDBJ/EMBL/GenBank |
|  | *Aspergilus oryzae* | DDBJ/EMBL/GenBank |
|  | Aspergillus clavatus | DDBJ/EMBL/GenBank |
|  | *Aspergillus flavus* | DDBJ/EMBL/GenBank |
|  | Neosartorya fischeri | DDBJ/EMBL/GenBank |
|  | *Emericella nidulans* | DDBJ/EMBL/GenBank |
|  | Ascosphaera apis | DDBJ/EMBL/GenBank |
|  | *Coccidioides immitis* | DDBJ/EMBL/GenBank |
|  | *Coccidioides posadasii* | DDBJ/EMBL/GenBank |
|  | *Rhizopus oryzae* | DDBJ/EMBL/GenBank |
|  | *Phycomyces blakesleenaus* | JGI DOE, http://genome.jgi-psf.org/Phybl1/Phybl1.home.html |
|  | *Mucor circinelloides* | The Mucor Genome Project, http://mucorgen.um.es/ |
|  | *Batrachochytrium dendrobatidis* | DDBJ/EMBL/GenBank |
|  | *Encephalitozoon cuniculi* | DDBJ/EMBL/GenBank |
|  | *Antonospora locustae* | Antonospora locustaeDB, http://gmod.mbl.edu/perl/site/antonospora01?page=general_help |
| *Entamoeba* spp. | *Entamoeba histolytica* | DDBJ/EMBL/GenBank |
|  | *Entamoeba invadens* | DDBJ/EMBL/GenBank |
|  | *Entamoeba dispar* | DDBJ/EMBL/GenBank |
| Metamonada | *Trichomonas vaginalis* | DDBJ/EMBL/GenBank |
|  | *Giardia intestinalis* | DDBJ/EMBL/GenBank |
| Trypanosomatidae | *Trypanosoma brucei* | DDBJ/EMBL/GenBank |
|  | *Trypanosoma cruzi* | DDBJ/EMBL/GenBank |
|  | *Leishmania major* | DDBJ/EMBL/GenBank |
|  | *Leishmania braziliensis* | DDBJ/EMBL/GenBank |
|  | *Leishmania infantum* | DDBJ/EMBL/GenBank |
| Chloroplastida+  Rhodophyta | *Arabidopsis thaliana* | DDBJ/EMBL/GenBank |
|  | *Populus trichocarpa* | DDBJ/EMBL/GenBank |
|  | *Vitis vinifera* | DDBJ/EMBL/GenBank |
|  | *Oryza sativa* | DDBJ/EMBL/GenBank |
|  | *Sorghum bicolor* | JGI DOE, http://genome.jgi-psf.org/Sorbi1/Sorbi1.home.html |
|  | *Selaginella moellendorffii* | JGI DOE, http://genome.jgi-psf.org/Selmo1/Selmo1.home.html |
|  | *Physcomitrella patens* | DDBJ/EMBL/GenBank |
|  | *Ostreococcus lucimarinus* | DDBJ/EMBL/GenBank |
|  | *Ostreococcus tauri* | DDBJ/EMBL/GenBank |
|  | *Micromonas* sp. RCC299 | JGI DOE, http://genome.jgi-psf.org/MicpuN2/ MicpuN2.home.html |
|  | *Micromonas pusilla* CCMP1545 | JGI DOE, http://genome.jgi-psf.org/MicpuC2/ MicpuC2.home.html |
|  | *Chlamydomonas reinhardtii* | DDBJ/EMBL/GenBank |
|  | *Volvox carteri* | JGI DOE, http://genome.jgi-psf.org/Volca1/Volca1.home.html |
|  | *Cyanidioschyzon merolae* | DDBJ/EMBL/GenBank |
|  | *Galdieria sulphuraria* | The *Galdieria sulphuraria* Genome Project, http://genomics.msu.edu/cgi-bin/galdieria/blast.cgi |
| Chromalveolata | *Tetrahymena thermophila* | DDBJ/EMBL/GenBank |
|  | *Paramecium tetraurelia* | DDBJ/EMBL/GenBank |
|  | *Cryptosporidium parvum* | DDBJ/EMBL/GenBank |
|  | *Cryptosporidium hominis* | DDBJ/EMBL/GenBank |
|  | *Toxoplasma gondii* | DDBJ/EMBL/GenBank |
|  | *Eimeria tenella* | WTSI, http://www.sanger.ac.uk/Projects/E_tenella/ |
|  | *Plasmodium falciparum* | DDBJ/EMBL/GenBank |
|  | *Plasmodium vivax* | DDBJ/EMBL/GenBank |
|  | *Plasmodium yoeli* | DDBJ/EMBL/GenBank |
|  | *Theileria annulata* | DDBJ/EMBL/GenBank |
|  | *Theileria parva* | DDBJ/EMBL/GenBank |
|  | *Babesia bigemina* | WTSI, http://www.sanger.ac.uk/Projects/B_bigemina/ |
|  | *Babesia bovis* | DDBJ/EMBL/GenBank |
|  | *Perkinsus marinus* | DDBJ/EMBL/GenBank |
|  | *Phytophthora sojae* | DDBJ/EMBL/GenBank |
|  | *Phytophthora ramorum* | DDBJ/EMBL/GenBank |
|  | *Phytophthora infestans* | DDBJ/EMBL/GenBank |
|  | *Hyaloperonospora parasitica* | VBI Microbial Database, http://phytophthora.vbi.vt.edu/ |
|  | *Thalassiosira pseudonana* | JGI DOE, http://genome.jgi-psf.org/Thaps3/Thaps3.home.html |
|  | *Phaeodactylum tricornutum* | JGI DOE, http://genome.jgi-psf.org/Phatr2/Phatr2.home.html |
|  | *Aureococcus anophagefferens* | JGI DOE, http://genome.jgi-psf.org/Auran1/Auran1.home.html |
|  | *Emiliania huxleyi* | JGI DOE, http://genome.jgi-psf.org/Emihu1/Emihu1.home.html |

### 4. Corrected and newly assembled sequences of CPRas proteins:

>Acanthamoeba castellanii CPRas, gene model based on the genomic scaffold Contig18902_Contig6945_Contig21178

MGVESAKHRLVLLKWCKAKAKSALEQEQKAINKKKKEKEKEKEKETTTIKLGISLKAKSKEPVVVAEEKEKSKRKRKGSAKPAKPKAKPKAQAQATNDEGGESGALAFGSELVYAGPEEMEDEDFQELDLDSSMASLARKNWETTDDGQTCMLDMLDTAGQEEYSCMRDQYMRVGQCFIVMYSITSRNSFDEARNIRDLILRIKDVDDVPIVLIGNKVDLEGDQREVGRNEGLEYARSCGMPFFESSAKTRVNVDEAVHELLRITPRTGVEYKLVVCGSGGVGKSAFTVQFISNHFVDCYDPTIEDSYRKQIVVSGLPVQNAAAAGRKTPKKSGFFGKVMDMFGGGSSSSSSSSSVTTAAKPPPAVRACVRSCVLLTRRGGLWFHRHTGLIVFCMDISGSMCVSTEVPALQNEWKALRQGGGKVTAHAHAHTRTHTRSTHVTHHMMRAQSKEEKNKEINASFNAENSYQYMPRENRNASYISRLECMQAAVDTHLQRLKVQSPNKKVVLITFNNEVTIVGDGSSGGKVVTGDRLGEFDDLLKVGHDLDTKALRPIAESLDDVSQKVAQLQENGATALGPALLLAVAIASHSRRSEVVVCTDGLSNVGVGSLEGAEKERGAEFYTRVGGLAKKNDTTINVLSIEGSDCAMDCLSRCAEMTSGTVNIVNPLELVRQIRVISQNPVIATNVKTSLFAHRHVLVADPRARSYRYLGWRALPTAEKTKKKAKKERKAVTPFFEYDVGNAMADTDLTFTYAIKEEMATELKAAPFQVQIHYTCKDGMQCMRVITATRRVTTKRDKTEAKADVAVLALASVQRAAREAQVGRFMEARMNLFASERLMKRAAHTDTQMEEHANFLGLTDQLDYELRQCLRRVGTTSGASGGKGKAKAREEDALEGLGDTTAKTLFKMRTSSKKEFLSGRRKKAVIDARRNDDATLRDMYYAKKF*

>Amphimedon queenslandica CPRas, assembled from WGS sequencing reads (starting from the read ti|930460968)

MDFGSDYVYAQEDNDEFEYVEESKFNHVTTGYSSVTADYNDDGETCLLDILDTAGQEEYSSMRDQYVREADCFIIVYSITDSSTFDEAKAMYDWTGRIRDKAMPAVLCGNKCDLEMSRSVSQKQGADYAAQIGIPFFETSAKDDVNIQEAICELIRRTPRARGKEYKVVTLGSGGVGKSSITIRFIQNHFVDQYDPTIEDSYRKQMVIKGIPDEMRVGAAKSSKKKSKKAATNSLGGGGGGKKKGGLFGSLFGKKSSGATPAPPPIDDDDDEETASAAPKKKEEKKVKVRRSNPNAIVLQLGNLGTCTDPSAEAHYFCSNCNAAVNCLSELKTNDGVTSWKCEFCGQQNDNVPLTVAPTGNAIEYEIEPAPKAEEGDDEDKPDNEAEAAKGGKTKAAPISQRSNLIIFVVDISGSMNITTEVPQLQAEWTSAAGRGGGNRHISRLECLKEAISRHMDHLAATEPNNKVALVTFDNKVTYYGDCSTSPQQLDSGVLNDYHQLMDQGQRVGSDLSLRKLTESVDDLKSKVHGLSTCGTTALGPSLALCVGLASNYPSAEIILCTDGLSNVGIGNLDSGNPDRDFYTTMGEHAQASHTTVSILGIEGGQCAMDILSSCAAITNGTVNILHPLEMVRQIRLIAQNPTVATEVQVTYIMHPAIVLDHPSESSSVTLVRYMVGNATRESDLSITFKLEKSKLKDENVLPFQVQIRYRRKDGSKWLRILTQSRKVTNDHKTVEEASNVAVLGIAAVQRAAKLAKDGRLEEARENLHAIDQMIQRGAQSDQQQEEHAIYREQANLLDLQLMKGTSGDTAAKQLHVSKNAHMGQFLSGAAKRKLVQERKADEAVQQQYYAYKFQ*

>Branchistoma floridae CPRas, corrected version of jgi|Brafl1|125403 (allelic variant: jgi|Brafl1|133020)

MQFGSEFVYLRDHPDESEESVDEDEEVRAFSKRRNSGQDDGKTCMLDILDTAGQEEFSAMRDQYMRTGNAYILIFDVTSRGSLDQAGSIYEWLLKIKGADHVPAVLCGNKIDLSASRQVTAEEGRQVAQRHGLPYLETSAKTGQNVEEAFHEVIRQTQRDGKNYKLAMLGAGGVGKSSLCVRFVQGDFVEDYDPTIEDSYRKLITVDDIPQDKLQKRNGKPQKKAVRTKTKTKKKTKKSSGAVLSGLGLFGRLLGGRSAMRDSSDSSEEEQQQRNADSSDSSDGSSSEEEQQQLENSLADTETGAAKRQKKIVKRKKADGNVVLVSLSCLENETTVMPRAPPSCAECNAVLSHLSKISNGENGQPGGWHCHFCNTRNDGIEEKDIPTDGTVEYVLAPADTPTDDPSGASPGVIPGAQGVVVLCVDVSGSMGVTTRVPQLQAEWKAMRDKKAGKEYVTRLECMTAAIARHLERLELDTPDRQVAIVTFQSHVQLFGDGRYPPTDVTGDTLNSYDGLISTGRHLAEKFELQPIKDSLATLKEKVCDLSAGGTTALGPALAVCAGVVADKPRSEIILCTDGAANVGVGDVKKDPGFYKKIGDFARSHKIVISIIGIEGENVALEQVSAAAATSGGTVNILHPLELVRQIRQIAQNNLVATDVRVTLMVHPALLVERNDQERKDGEVSHRIEVDVGNATKSTDMTFSIRQRASNIDTNMAAFPAQAQISFTRKDGARCLRVITMETKATQNREDMEKNLNIAVTGLAAVQKTSALAEGGKNDHAKVHLQNVERLLARGSVGDSQKEEHYIFLEENRELAKELSEPRRGPQTGASADQRFKNLYKKKHASVEQFLXGKKKGKAVKGRITDKKLNQQYYDYEYQPEEEE*

>Capsaspora owczarzaki CPRas, assembled from WGS sequencing reads (starting from the read ti|1984356590)

MFFASRYVHATSSAAPLFEDIDASLEEQDLVTVSRRNRVDDGETCLLDILDTAGQEEYSSMREQYMRVGDCFLVVYSVTSRSSFDSACLMRDFILRIRDTDEIPVLVGNKCDLASDREVTREEGLAAAKRMGAAAFFETSAKKRLNVEESFFELVRHTPRQGVEYRVVVLGDGGVGKSALTIQFIQHHFVDEYDPTIEDSYRKQCVISGLRHAAPSARRAAGRPKSKCKLSASAAGLTLTNTTTYIQSKLTFPNLFGCFPLDCWMIHCHSGFAGTFQLLCVQGAEIMSTNVGMLSLGSLAEPAEIMTGDPVKCAGCHGVLSSLSTLTLSNKSAKQAVWKCEFCGHENANIHAGEVPDQPTVDYILESVAPSMSQSQDTSLVVYCLDISGSMCVTTELPRLQSEWRAARTTTGSDAPVSADQYLPGETRGAKYMSRLQCIQEAVSRNLQRIHDENPNRRVVLVVFSSEVVVIGDGLTHIPETVTGSKLESFATLTDAGHSLAEKVEPISKSLAQLKRKVEGLEESGATALGPALAVAVGMAGRVPGSEVVLCTDGIPNVGLGAIDGAADPLAAAAFYTRIGELAREQDTTISLLGISGTDCGLQHLSACAKMTSGTVNVLHPVEIVREMRKISQNPIVANNVEITLRSSPGIQFDGVNAPESGSTAVIKLGNALRECDRAFMFRNIVPAKQLGAHTTFQAQIRFTRLDGAKCLRVITARREITSDVEVARNGLVISTMSLAAAQLGSVLAQNAAFSQARALLSGTERLMRVCAKTDAQMEEHATFVGETHDLDQAISEYSRKSRKALATPGKADTAEMSSVLFGTAKSTRETKLPDGIVRTLATMSTTPVNLFYSGEAKRKLVTSRKTTEAAKREYYAYTC*

>Monosiga brevicollis CPRas, corrected version of jgi|Monbr1|20657

MALHFGGEFFSPATTSPAIIKVPDAFHISVNSAKHDTSAPLLTQSDSCLLDILDTAGQEEYSSMRDMYCQTGDAFAIVFSVTSRASFDEARLMKDWVLRVRDVDTTDQVALILIGNKSDLNVAREVSSTEGQRLADEWHCTYIETSAKTGFNVNRAFEELVLSFARLNPRMTQLKTVVMGSGGVGKSALTVQYIQHVFVECYDPTIEDSYRAQRQIPGLAAVRQAFRHKKAGQTKSKNGCFNWSILGRRRSKEQNQGSNGNASPKTEPIATKLVANPPLNTMLVRHRHDAGLEGQTGGRRMVSILTKCLFLPAVRQVSLGAITQGFEPVDTMPFQCKACSAYMVDACVDEARDKNNAEMLRCRICQNLSDVPASYAVLAREAVDLVLDDPSSNTDAATQKPTDKSEIVAHKDNRLMVLAMDISGSMDVEVTVASLQAEWAAMRDNGRRRHLTRLDCMKEAMCRRLDYLAAQYPERRMILVTFENDVQVYGTPEEAPLIIESGDDMLHLQATACLQAQKAGWQLPTIGQSLEAWKLKIQNLATKGSTALGPALCVSVGLVEALAGAGVSEIILCTDGQPNRGVGRLDYRTKSSTFYEAIGEQARSHSSSISVIGTTGVSCELGIVGACAEASGGSITTLDPLELVRYMRELVQNAAVASDVKVRVCLPSTVQLEVDHRLLPTTSGGTSIDVGNVYRNADMGFALGGSFEKMPPGSAVQVQIAFTDLEGRRCLKVLTRRLPRLATTAREMGEHLDLAVQATVGVQRAARLGLAGHGPSGYELLIGLKHVLETSKEGNKMDEEVYSFVAESQSLAQQLHRLQPGQVQLSDDAVRELRHMKSAPLAQFVSAERKVDQTKARQTTQEVASKYYNYRF*

>Naegleria gruberii CPRas, corrected version of jgi|Naegr1|68720

MSLQLATKTLYHNKKKTDQQEDEEYDFFPLRLLEESHSLKKAYTHSRRKQLGYSSVGGESNTTGGSDHVALIDLLDTAGQEEYSSLRDQYYRTSYGFLIVFSITDSSSLEDCQTIWEQILRTKDVDSFPCVLIANKSDLEEERIVSKQQVKEVALRLGIPYFETSAKQRINVDEPIHELVRMCGRLDEYRFSIVGSGGVGKSAYCTQFIQNVFVEQYDPTIEDSYRKQIVVSNVPLDDAQSSSVSSSTSKKKSSGGGFLSRLFKKSEKKTASTVSQACSSSIDLSNEQTIKHPKSDANVAALCMNGLLAECPKALGPLVTCSNCKAALNGEKKRNHWKCSFCDTENVLPSNVSSIPEMIEHKLEESSVNDDDGLLVFCIDISGSMCQSEQLPSYSVLNLDLRQSDQQEKNRRLLRELGLESEVSVHNQFLPNESRSSSYVSRMDCLKMALDIQVTETARESPNKRIAIVTFSSNVQIIGDARSQTRASVPAVLLDNFEGLFKLGQSYRTSILNNVSLANDNLIEYIYSIAEDGATALFPALLVSLGIASQQKGSKIILCTDGVATTGLAGISEEISKRAKSIKDQEECCTKIANLSCEYGININVFGVEGSNCNLSLIGKLADNTGGLTNIVKPVELRRQMRQIIDNPVIATDVKVQMLFSNPISPKNQEVSKLDKYSMVTQSISSATAETDVTIEMEVKDDKKDKGLIQAQIYYTASSNGCKYVRVVNQPVKITSDRSVSLENVDVSVLSLNAVLQSSILAESGKIRDARLKLLSMNKLLEEVSKSYGQQEEMNAFVNNTQDLDEDLRQCENDSSMRETDKLSSLLYRMKKSNRTAFLAGERKREIVQTRKKHTGQSNNNNQSSSSNSGITPSNSRIQTEDPSIFTNRDWLAEEERIPEPANISQITTPTDHHHEQQEENNHCIVCMDKEINVVLVPCGHMIMCDGCANKLTNKSCPTCRKPITQIVKVFK*

>Strongylonectrotus purpuratus CPRas, corrected version of XP_797936.2

MDFGSKYVFVKREEEEEEEVLDDAEVSTGLDNLKVEIQADSSEDGEPCMIDLLDTAGQEEYSALRDQYMRTCQGLAIVYSITDLASFEEAIKIYQFGLSIKGEDHIPAVLIGNKNDLESERKVSAEKGKEAARKYNMVFYETSAKTGHNVQESLKALVRETPRYGSRYKFVVVGGGGVGKSSITVRFVQDIFVETYDPTIEDSYVKQIVVSGIPADKLNPPPKPQASSVPNAPPPPPQAQRVSKSFGLFKRSKKQKKSEFTAPMMNACCSMGPPLGNSSFLQTKPSHSKSKGASKVAAPKSYRRADANVILLSLGSLANDPVIMTGDPVRCSKCNILLSHVSRLDPVENEDKRNWICEFCGQQNEGLEMVDEEVPKENSVDYLLAAAPEKAAEGEKKDETIKPSSTPGLVLYCVDMSGSMAERTQLPELQAAWMAQRGQRNSSVTRLECMKQAITRQLDFYELDEEIEKRIGLVTFESQVVARGDGSQTTRSVSEPMADMNALIQQGKDLASHFNIRPVSTAVGDLKTQVSNLRTAGSTALGPALCVCVGLLAKEPGSEIVLCTDGQPNVGVGSFGYSSRGDNPEAISFYRQIGNIAKEQQTTISIIGIQTNQSLQMEHISNTVSITGGNLNLLDPHELTRQIRQLGQDPVVANDVELTLVLHPTLQFDESMTEGVIQDGNRLTLNMGNVHQSTDLTFRFKLKQGQDTTQQNKLPFQVEIRYTKTDGRRFLRTITQERDTTSDRSTMETEMNPTVTGLATVQEAAKMGEKGDKSKARGHMWAMAKMMQRSNASPGSTSLQEQRSRSEGTHQFLNQAAELDRVYDEDSDDDAVAATSNMYRNAKADKFRSAEAKTSGDNMRRKKGKGSAFSKQYYDYK*
